# Loss of Synaptic Connectivity, Particularly in Second Order Neurons Is a Key Feature of Diabetic Retinal Neuropathy in the Ins2^Akita^ Mouse

**DOI:** 10.1371/journal.pone.0097970

**Published:** 2014-05-21

**Authors:** Jose R. Hombrebueno, Mei Chen, Rosana G. Penalva, Heping Xu

**Affiliations:** Centre for Experimental Medicine, School of Medicine, Dentistry and Biomedical Sciences, Queen's University, Belfast, United Kingdom; Queen's University Belfast, United Kingdom

## Abstract

Retinal neurodegeneration is a key component of diabetic retinopathy (DR), although the detailed neuronal damage remains ill-defined. Recent evidence suggests that in addition to amacrine and ganglion cell, diabetes may also impact on other retinal neurons. In this study, we examined retinal degenerative changes in Ins2^Akita^ diabetic mice. In scotopic electroretinograms (ERG), b-wave and oscillatory potentials were severely impaired in 9-month old Ins2^Akita^ mice. Despite no obvious pathology in fundoscopic examination, optical coherence tomography (OCT) revealed a progressive thinning of the retina from 3 months onwards. Cone but not rod photoreceptor loss was observed in 3-month-old diabetic mice. Severe impairment of synaptic connectivity at the outer plexiform layer (OPL) was detected in 9-month old Ins2^Akita^ mice. Specifically, photoreceptor presynaptic ribbons were reduced by 25% and postsynaptic boutons by 70%, although the density of horizontal, rod- and cone-bipolar cells remained similar to non-diabetic controls. Significant reductions in GABAergic and glycinergic amacrine cells and Brn3a^+^ retinal ganglion cells were also observed in 9-month old Ins2^Akita^ mice. In conclusion, the Ins2^Akita^ mouse develops cone photoreceptor degeneration and the impairment of synaptic connectivity at the OPL, predominately resulting from the loss of postsynaptic terminal boutons. Our findings suggest that the Ins2^Akita^ mouse is a good model to study diabetic retinal neuropathy.

## Introduction

Diabetic retinopathy (DR) is a sight-threatening complication of diabetes and the leading cause of blindness in working populations [1]. Neural retinal degeneration together with microvascular dysfunction, constitute the key pathologies of DR [2], [3]. Vascular complications (i.e. diabetic retinal vasculopathy) comprise a well-defined set of changes, which progress from capillary degeneration and vascular leakage in non-proliferative DR to retinal neovascularization in proliferative DR [4], [5]. Features of diabetic retinal vasculopathy including microaneurysms, intraretinal hemorrhages and neovascularization are readily detectable in fundus examination. Visual impairment in DR is often associated with vascular dysfunction (macular oedema, vitreous hemorrhage or tractional retinal detachment). As a result, DR-related vasculopathy has been researched extensively. Although diabetes related retinal neural degeneration (diabetic retinal neuropathy) has been recognized for over five decades [6], [7], the detailed neural pathology remains ill-defined. With advancements in the management of diabetic macular oedema and proliferative DR, further vision improvement will rely upon the development of neuroprotective strategies. From this perspective, a clearer understanding of how the neurosensory retina is affected under diabetic conditions will be crucial.

Diabetic retinal neuropathy has been investigated in humans and various animal models. The majority of studies have reported a progressive loss of inner retinal neurons, including amacrine and retinal ganglion cells (RGCs) [8]. This degeneration is likely to be responsible for the early electrophysiological deficits in DR [9], [10]. A growing body of evidence suggests that neuropathy in DR may also involve other neural elements. For example, impairment in colour vision (in particular blue-sensitive defects) and decreased contrast sensitivity constitute an early feature characterizing visual dysfunction in DR [11], [12]. Studies in animal models have revealed some cellular components of diabetic retinal neuropathy. In diabetic rodents for example, photoreceptor death [13], [14], reduced expression of presynaptic photoreceptor proteins [15], abnormalities in horizontal and bipolar cell synaptic terminals [13], [16] and dendritic remodeling of RGCs [17] were observed.

In the present study, we characterized retinal neuropathy at different stages of disease in the Ins2^Akita^ mouse (model of type-1 diabetes). Previous studies have shown that Ins2^Akita^ mice develop various early DR-related retinal abnormalities, including increased vascular permeability, RGCs apoptosis, reduced retinal thickness [18], [19] and abnormal electroretinographic responses [20]. We found that the Ins2^Akita^ mouse presents a variety of retinal neuropathic lesions including the degeneration of cone photoreceptors after a relatively short disease duration (3-month old), and loss of synaptic structures in second order neurons and reduced number of amacrine cells and RGCs at the later stages of the disease (9-month old).

## Materials and Methods

### Animals

Male heterozygous Ins2^Akita^ mice (Originally purchased from Jackson Laboratory, Bar Harbor, USA, stock number 003548) of C57BL/6J background and age-matched non-diabetic siblings were used in the study. The Ins2^Akita^ mouse develops robust hyperglycemia by 4.5 weeks of age [21]. Only males were used in this study. Experimental mice were generated by mating Ins2^Akita^ heterozygous males with C57BL/6J females, and diabetes confirmed in 6-week old littermates by blood glucose measurement (>250 mg/dl; the majority of Ins2^Akita^ mice had ≥540 mg/dl – above the maximal detectable level of the glucometer; non diabetic siblings had 151±19.8 mg/dl). All mice were housed in standard pathogen-free animal housing rooms with 12/12-hour light/dark cycle and free access to food and water. All procedures were conducted under the regulation of the UK Home Office Animals (Scientific Procedures) Act 1986 and approved by the Ethical Review Committee of Queen's University Belfast (Project Licence Number: PPL 2664). The study was conducted in compliance with the Association for Research in Vision & Ophthalmology Statement for the Use of Animals in Ophthalmology and Vision Research.

### Electroretinography

Scotopic electroretinogram (ERG) responses were evaluated in Ins2^Akita^ and control mice at 3, 6 and 9 months of age (n = 6 mice per strain/age). Mice were dark-adapted overnight and all procedures were conducted under dim-red light (<1 Lux). Briefly, mice were anesthetized with an intraperitoneal injection of ketamine hydrochloride (60 mg/kg; Fort George Animal Centre, Southampton, UK) and xylazine (5 mg/kg; Pharmacia & Veterinary Products, Kiel, Germany) and pupils dilated using 1% tropicamide and 2.5% phenylephrine (Chauvin, Essex, UK). The head was secured with a stereotaxic frame and the body temperature maintained with a homeothermic pad at 38°C (Kobayashi Healthcare, London, UK). ERG was recorded using mouse corneal ERG electrodes in response to single white light flash, delivered by a standard Ganzfeld Stimulator (LKC Technologies, Gaithersburg, MD, USA). ERG signals were bandpasss filtered between 0.3–500 Hz (without notch filtering), amplified 500-fold and digitized at 2 kHz by using the CMGS-1 electrophysiology system (LKC Technologies). For each animal, 8 light intensities were used, ranging from 0.008 cd·s/m^2^ to 25 cd·s/m^2^. ERG signals were averaged from 5 responses at each intensity level, with an inter-stimulus interval of 10 seconds (0.008, 0.025 cd·s/m^2^), 15 seconds (0.08, 0.25, 0.8 cd·s/m^2^) or 30 seconds (2.5, 8, 25 cd·s/m^2^). Oscillatory potentials (OPs) were obtained by a low band filtering at 75 Hz and recorded using 25 cd·s/m^2^ flash intensity. The following ERG parameters were measured: a-wave amplitude (baseline to negative a-wave peak), b-wave amplitude (a-wave peak to positive b-wave peak), a-wave implicit time (from stimulus onset to negative a-wave peak), b-wave implicit time (from stimulus onset to positive b-wave peak), OPs amplitude (the summed amplitude of wavelets 2–5) and OPs implicit time (the summed implicit times of wavelets 2–5). The b/a-wave amplitude ratio was calculated at the highest flash intensities (0.8, 2.5, 8, 25 cd·s/m^2^).

Photopic ERG responses were also evaluated in Ins2^Akita^ and control mice at 3 and 9 months of age (n = 5 mice per strain/age). Three light intensities (2.5, 8 and 25 cd·s/m^2^) were used to stimulate the light-adapted mice (on a 30 cd·s/m^2^ background light). The amplitude and implicit time of the photopic b-wave were averaged from 5 responses at each light intensity level.

### Clinical investigations

Animals were anesthetized by isoflurane inhalation (Merial; Animal Health Ltd.; Essex, UK) and pupils dilated as described above. A topic endoscopic fundus imaging system was used to obtain fundus images as described previously [22], [23]. Spectral Domain Optical Coherence Tomography (SD-OCT) was conducted using the Spectralis Heidelberg OCT system (Heidelberg Engineering, Heidelberg, Germany).

### Retinal thickness analysis

Retinal thickness was measured in Ins2^Akita^ and control mice at 3, 6 and 9 months of age (n = 6 animals per strain/age) from the SD-OCT images. Three types of measures were conducted: 1) total retinal thickness: from the nerve fibre layer (NFL) to the out margin of photoreceptor outer segments (OS); 2) inner retinal thickness: from the NFL to the margin between the inner nuclear layer (INL) and outer plexiform layer (OPL); 3) outer retinal thickness: from the INL/OPL margin to the out margin of OS. The SD-OCT layers were defined according to previous investigations in the mouse retina [24], [25]. Retinal thickness was measured at 300, 600 and 900 µm eccentricities from the optic disc (OD) in dorsal-ventral and nasal-temporal sectors. Isodensity maps of average retinal thickness were constructed using Sigma Plot v12.0 (Systat Software, London, UK).

### Immunohistochemistry

Mice were sacrificed by CO_2_ inhalation and eyes dissected and fixed in 2% paraformaldehyde for 2 h. Eyes were then processed for immunohistochemistry as previously described [26], [27]. Briefly, 14 µm-thick retinal cryosections were incubated overnight (4°C) with primary antibodies (Table 1) diluted in 0.5% TritonX-100 with 10% normal donkey serum in PBS. Sections were then incubated for 2 h at room temperature with appropriate fluorophore-conjugated secondary antibodies. All sections were cover-slipped with Vectashield-DAPI (Vector Labs, Burlingame, CA) and examined by confocal microscopy (C1 Nikon Eclipse TE200-U, Nikon UK Ltd, Surrey, UK).

**Table 1:** Primary antibodies used in the study.

Antigen	Antiserum	Dilution	Source	Retinal localization
Rhodopsin	mouse-rhodopsin, clone 4D2	1:500	Chemicon	Rod photoreceptors
Cone arrestin	rabbit anti-cone arrestin	1:10000	Chemicon	Cone photoreceptors
Bassoon	mouse anti-bassoon	1:400	Enzo	Synaptic ribbons
Secretagogin	sheep anti-secretagogin	1:400	Biovendor R&D	Cone bipolar cells
PKCα	rabbit anti-PKCα	1:500	Santa Cruz	Rod bipolar cells
Calbindin	rabbit anti-calbindin	1:1000	Chemicon	Horizontal cells
γ-aminobutyric acid (GABA)	guinea pig anti-GABA	1:500	Chemicon	GABAergic amacrine cells
Glycine Transporter-1(GlyT1)	goat anti-glyT1	1:3000	Chemicon	Glycinergic amacrine cells
Brn3a	goat anti-Brn3a	1:500	Santa Cruz	Retinal ganglion cells

### Retinal morphometry

Confocal images were used to quantify retinal neurons and synaptic structures in Ins2^Akita^ and control mice at 3, 6 and 9 months of age (n = 3 animals per strain/age). Data was acquired from 3 sections per eye at the same retinal eccentricities (0.6, 1.2 and 1.8 mm from the OD). Confocal settings remained unchanged in all studies during image acquisition. Images were analysed using ImageJ software (National Institutes of Health, Bethesda, MD).

Using the listed panel of antibodies we carried out the following analyses: 1) rod and cone photoreceptor density (rods estimated as the difference between DAPI^+^ nuclei at the outer nuclear layer and the number of cone somata), 2) photoreceptor synaptic ribbon density, 3) horizontal, rod-bipolar and cone-bipolar cell density, 4) horizontal and rod-bipolar cell dendritic spines density, 5) rod- and cone-bipolar dendritic length (measured from the apical border of the cell soma), 6) rod- and cone-bipolar cell axon bouton density, 7) GABAergic and glycinergic amacrine cell density, and 8) RGCs density. At least 20 retinal images per strain/age were analyzed and values averaged and normalized to 100 µm retinal length. The density of dendritic spines in horizontal cells was measured in 4 cells per image. The density of dendritic spines and axon boutons in rod-bipolar cells and the dendritic length of rod- or cone-bipolar cells were measured in 20 cells per image.

### Data analysis

ERG responses (scotopic a-wave, b-wave and photopic b-wave), and morphometry data were analysed using 2-way ANOVA (two strains of mice and different ages), followed by post-hoc Bonferroni's pairwise comparisons. OPs responses and retinal thickness were analysed using unpaired Student's *t*-test. Data were expressed as mean ± SEM (ERG) or ± SD (retinal thickness, retinal morphometry). *P*<0.05 was considered statistically significant.

## Results

### Abnormal ERG responses in the Ins2^Akita^mouse

**Figure 1:**
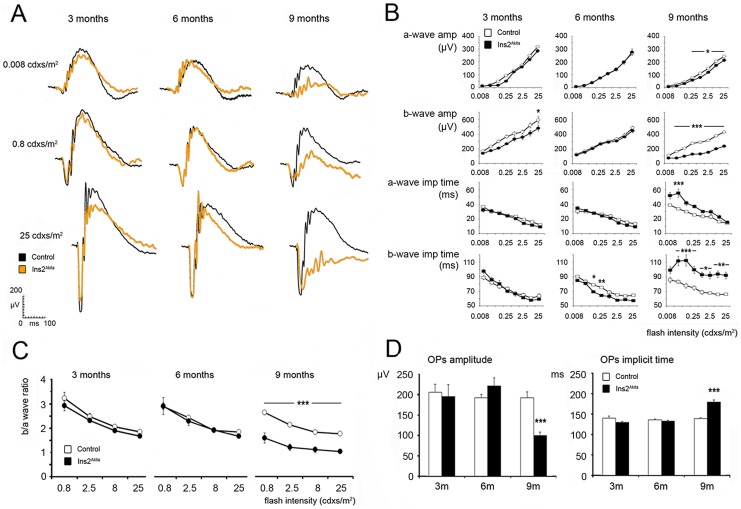
Abnormal scotopic electroretinogram (ERG) responses in the Ins2^Akita^ mouse. (A) Representative scotopic ERG responses obtained from control (black) and Ins2^Akita^ mice (orange) of different ages. (B) The amplitude (µV) and implicit time (milliseconds, ms) of the dark adapted a- and b-waves in control and Ins2^Akita^ mice. (C) The b/a-wave ratio in different ages of control and Ins2^Akita^ mice. (D) The amplitude and implicit time of oscillatory potentials in control and Ins2^Akita^ mice. (B-D) n = 6 mice per strain/age, *P<0.05, **P<0.01, ***P<0.001. 2-way ANOVA (a- and b-wave responses), Student's *t*-test (OPs responses).

Scotopic ERG analysis in 3-month old Ins2^Akita^ mice showed a slight but statistically significant reduction in the b-wave amplitude in the higher flash intensity (25 cd·s/m^2^) compared to age-matched controls (Figure 1A-B). Interestingly, 6-month old Ins2^Akita^ mice had significantly faster b-wave responses (0.08 and 0.25 cd·s/m^2^; b-wave implicit time, Figure 1B) compared to control mice. By 9-month old, the Ins2^Akita^ mouse displayed smaller amplitude and increased implicit times in both a-wave (reduced amplitude between 0.8–25 cd·s/m^2^ flash light; increased implicit time at 0.025 cd·s/m^2^ flash light) and b-wave (reduced amplitude between 0.025–25 cd·s/m^2^ flash light; increased implicit time between 0.025–25 cd·s/m^2^) responses. Further analysis using b/a-wave ratio revealed a strikingly disproportionate reduction in the b- and a-wave amplitudes (p<0.001; Figure 1C), suggesting that the abnormal drop in b-wave amplitude may arise from disorders at the distal retina rather than from photoreceptors.

OPs responses were evaluated to assess the functional activity of amacrine cells (Figure 1D). No changes were observed in the amplitude or implicit times of OPs by 3 months of age. An apparent increase in the OPs amplitude was evident in 6-month old Ins2^Akita^ mice, but failed to reach statistical significance. Closer inspection of these traces, however, revealed an increase in the fourth OP wavelet (p<0.05; figure S1A). OPs implicit times appeared normal at this stage (Figure 1D). By 9-month old, OPs responses were significantly reduced and delayed in the Ins2^Akita^ mouse (p<0.001; Figure 1D and figure S1B).

No differences were observed in the photopic b-wave amplitude between Ins2^Akita^ mice and sibling controls of 3 months old (figure S1C). However, by 9 months of age, the amplitude of the photopic b-wave was significantly reduced in 25 cd·s/m^2^ flash light (p<0.01; figure S1D). The implicit time of the photopic b-wave remained similar to age-matched controls in both age groups (data not shown).

### Retinal degeneration in the Ins2^Akita^mouse

**Figure 2:**
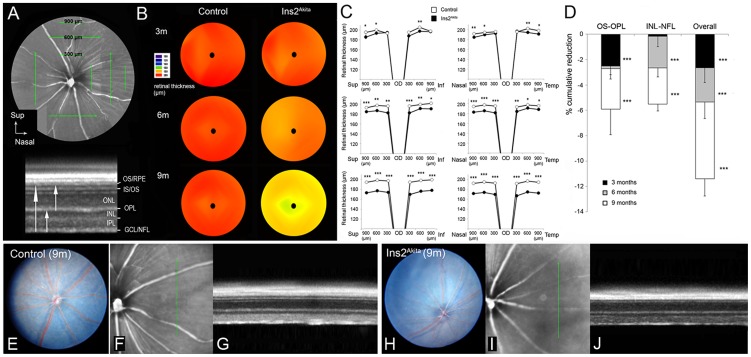
Retinal degeneration in the Ins2^Akita^ mouse. (A) SD-OCT image from 9-month old control retina. Retinal thickness was measured at 300, 600 and 900 µm eccentricities in dorso-ventral and nasal-temporal retinal regions, from NFL-OS (total retinal thickness), NFL-INL (inner retinal thickness) and OPL-OS (outer retinal thickness). Isodensity maps (B) and quantitative analysis (C) of total retinal thickness from control and Ins2^Akita^ mice at different ages. (D) The cumulative reduction of outer, inner and total retinal thickness in different ages of control and Ins2^Akita^ mice. Fundus (E, H) and OCT images (F-G, I-J) taken from 9-month old control (A-C) and Ins2^Akita^ mice (D-F). (B-D) n = 6 mice per strain/age, *P<0.05, ** *P*<0.01, *** *P*<0.001 compared to control mice of the same age. Student's *t*-test. RPE, retinal pigment epithelium; IS/OS, photoreceptor inner/outer segments; ONL, outer nuclear layer; OPL, outer plexiform layer; INL, inner nuclear layer; IPL, inner plexiform layer; GCL/NFL, ganglion/nerve fibre layer.

SD-OCT was carried out in 3-, 6-, and 9-month old control and Ins2^Akita^ mice (Figure 2). As shown by isodensity maps, there was an age-dependent reduction in the overall retinal thickness (OS-NFL) of Ins2^Akita^ mice but not age-matched controls (Figure 2B). This reduction occurred in all retinal sectors for all the eccentricities analysed (Figure 2B-C). Further analysis of outer (OS-OPL) and inner (INL-NFL) retinal thickness revealed that at 3 months of age, only the outer retina was affected in the Ins2^Akita^ mouse (p<0.001, Figure 2D). From 3–6 months, no further reduction was observed in the outer retina, but a significant reduction was detected in the inner retina (p<0.001). From 6–9 months, the thickness of both outer and inner retina was further reduced (p<0.001) compared to non-diabetic controls.

Despite a significant reduction in retinal thickness, other human DR related lesions (e.g. microaneurysm, exudates) were not detected in fundus imaging and SD-OCT investigation up to 9 months of age in Ins2^Akita^ mice (Figure 2E-J).

### Characterization of diabetic retinal neuropathy in the Ins2^Akita^mouse

#### Photoreceptor cells

**Figure 3:**
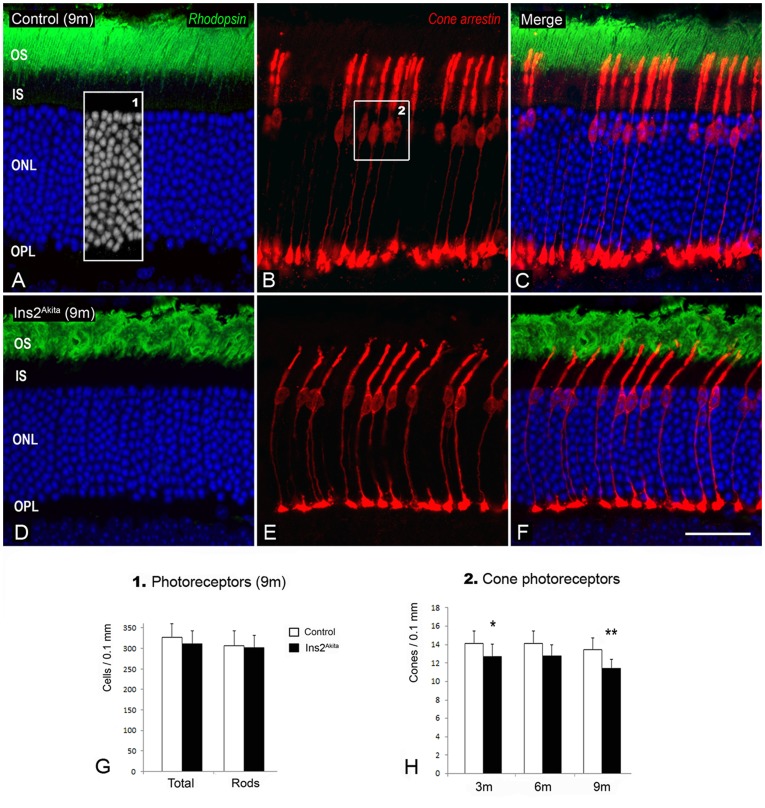
Cone photoreceptor degeneration in the Ins2^Akita^ mouse. (A-F) Retinal photomicrographs of rhodopsin (green) and cone-arrestin (red) immunoreactivities in 9-month old control (A-C) and Ins2^Akita^ (D-F) mice. (G-H) Cell densities of total photoreceptors (DAPI^+^ nuclei - *box1*), cones (*box2*) and rods (difference between DAPI^+^ nuclei and cones) in 3-, 6- and 9-month old control and Ins2^Akita^ mice. (G-H) n≥20 retinal images per strain/age, *P<0.05, **P<0.01 compared to control mice of the same age. 2-way ANOVA. OS, outer segments; IS, inner segments; ONL, outer nuclear layer; OPL, outer plexiform layer. Scale bar: 30 µm.

Rod and cone photoreceptors were evaluated by using antisera against rhodopsin and cone-arrestin respectively (Figure 3). Control and Ins2^Akita^ mice showed normal expression of rhodopsin in rod outer segments up to 9 months of age (Figure 3A-F). No significant changes were observed in the total number of photoreceptors and rods from 3-9 months between control and Ins2^Akita^ mice (Figure 3G). Interestingly, the number of cone photoreceptors was 10% less in 3-month old Ins2^Akita^ compared to age-matched controls (p<0.05) and further decreased by 9 months of age (p<0.01; Figure 3H). Thus, cone but not rod photoreceptors selectively degenerate from an early stage in Ins2^Akita^ mice.

### Outer plexiform layer connectivity

**Figure 4:**
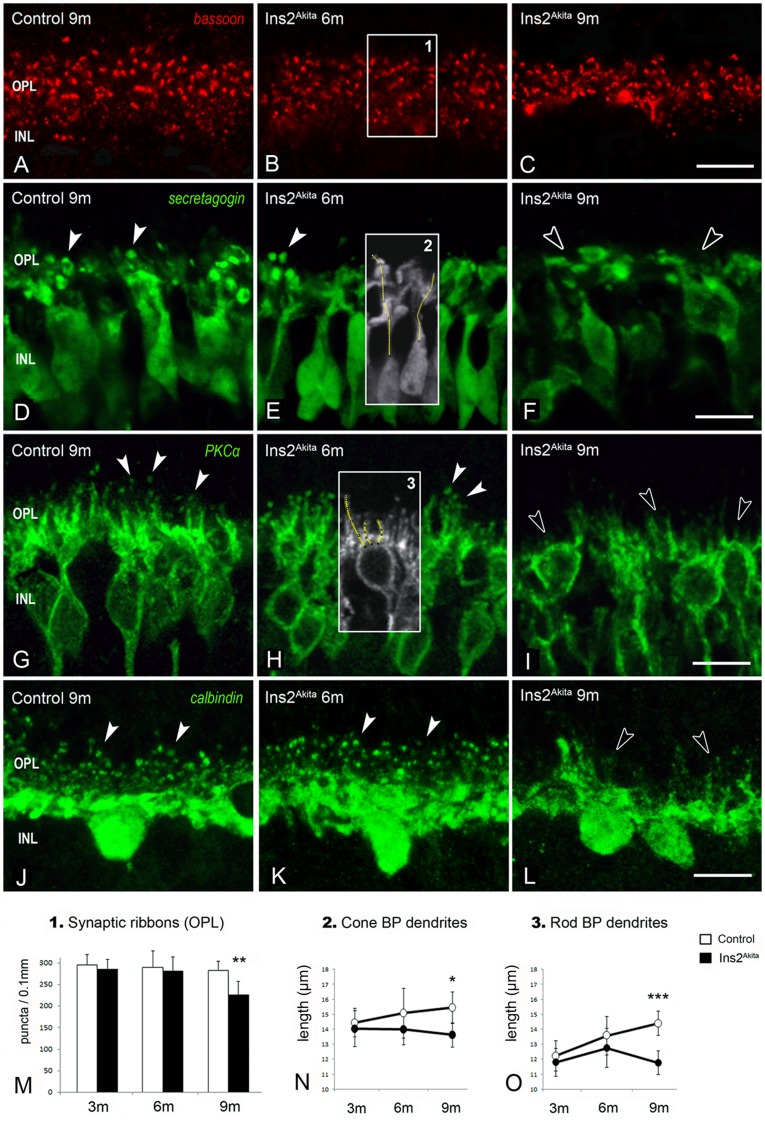
Synaptic elements in the OPL of control and Ins2^Akita^ mice. (A-C) Photoreceptor synaptic ribbons immunostained with bassoon in 9-month old control and 6- and 9-month old Ins2^Akita^ mice. Immunostaining of cone-bipolar (secretagogin) (D-F), rod-bipolar (PKCα) (G-I) and horizontal cells (calbindin) (J-L) in 9–month old control and 6- and 9-month old Ins2^Akita^ mice. Normal dendritic arbors of cone-bipolar, rod-bipolar and horizontal cells at the OPL (arrowheads). Loss of dendritic complexity in cone-bipolar, rod-bipolar and horizontal cells in 9-month old Ins2^Akita^ mice (open-arrowheads). Quantitative analysis of photoreceptor synaptic ribbons (*box1*) (M) and cone-bipolar (*box2*) (N) and rod-bipolar dendritic length (*box3*) (O) in control and Ins2^Akita^ mice at different ages. (M-O) n≥20 retinal images per strain/age, *P<0.05, **P<0.01, ***P<0.001. 2-way ANOVA. OPL, outer plexiform layer; INL, inner nuclear layer. Scale bar: 10 µm.

**Figure 5:**
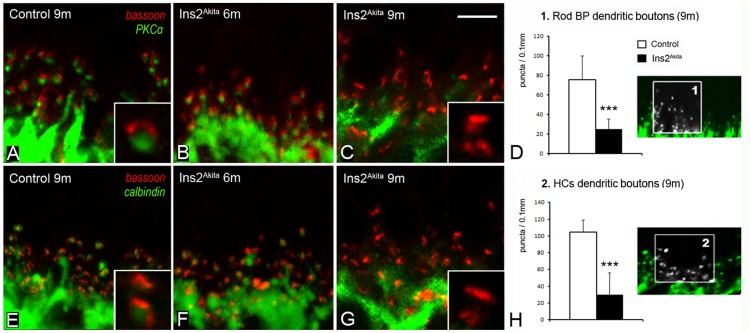
Synaptic structure in the OPL of control and Ins2^Akita^ mice. Photomicrographs of retinal sections double-labelled for bassoon (red) with PKCα (A-C) or calbindin (E-G) in 9-month control and 6- and 9-month old Ins2^Akita^ mice. Absence of paired connectivity between photoreceptor terminals and second-order neurons in 9-month old Ins2^Akita^ mice (insets C, G). Quantitative analysis of rod-bipolar (*box1*) (D) and horizontal cell (*box2*) (H) dendritic boutons in control and Ins2^Akita^ mice at different ages. (D-H) n≥20 retinal images per strain/age, ***P<0.001. 2-way ANOVA. Scale bar: 10 µm, inset 5 µm.

We next investigated synaptic connectivity at the outer retina by studying pre- and post-synaptic processes at the OPL (Figure 4, 5). Immunostaining of bassoon revealed synaptic ribbons in rod and cone cell terminals (Figure 4A). Many of the presynaptic ribbons displayed a typical horseshoe-like morphology in Ins2^Akita^ mice (Figure 4B-C). However, despite the absence of morphological anomalies, a 25% reduction of photoreceptor synaptic ribbons was detected in 9-month old Ins2^Akita^ (p<0.01, Figure 4M). This reduction did not correlate with a proportional loss of total photoreceptors at this stage (Figure 3G), suggesting additional damages to the photoreceptor synaptic complex.

Photoreceptor postsynaptic elements were identified by immunostaining of secretagogin (cone-bipolar cells) (Figure 4D-F), PKCα (rod-bipolar cells) (Figure 4G-I) and calbindin (horizontal cells) (Figure 4J-L). An age-dependent increase in the length of cone- and rod-bipolar dendritic projections (Figure 4N, 4O and figure S2) was observed in non-diabetic controls. By contrast, there was a significant reduction of in the length of both cone-bipolar (15%, p<0.05) and rod-bipolar dendritic projections (20%, p<0.001) in 9-month old Ins2^Akita^ mice (Figure 4N, 4O and Figure S2). The number of horizontal cell dendritic tips (postsynaptic boutons) was also reduced in this stage (Figure 4J-L).

Double immunostaining against bassoon and PKCα or calbindin showed the close association between photoreceptor presynaptic terminals and postsynaptic boutons of rod-bipolar or horizontal cells in control and 6-month old Ins2^Akita^ mice (Figure 5A-B, 5E-F). However, the absence of paired connectivity, predominately due to a lack of dendritic boutons in second-order neurons, was observed in 9-month old Ins2^Akita^ mice (Figure 5C, G). Quantitative analysis revealed a 70% reduction (p<0.001) of both rod-bipolar (Figure 5D) and horizontal cell dendritic tips (Figure 5H) at this stage compared to age-matched controls.

### Changes in bipolar and horizontal cells

**Figure 6:**
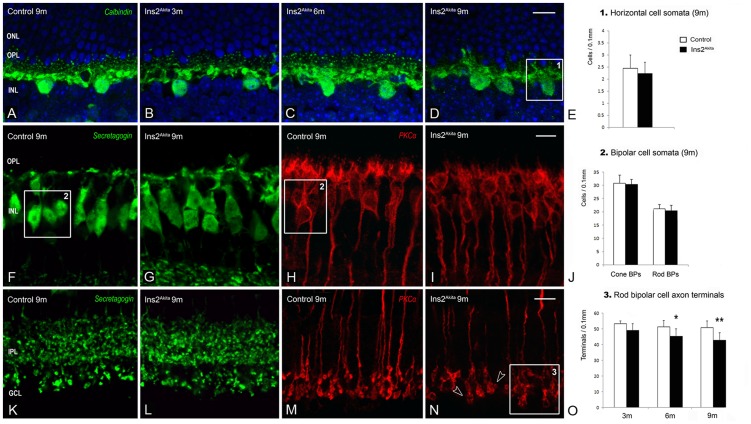
Second-order neuron changes in the Ins2^Akita^ mouse. Immunostaining and quantitative analysis of horizontal cell (*box1*) (A-E), cone-bipolar (*box2*) (F-G, J) and rod-bipolar (*box3*) (H-J) cell somata in control and Ins2^Akita^ mice at 9 months of age. (K-L) Cone-bipolar cell axon terminals in 9 month-old control and Ins2^Akita^ mice. (M-O) Immunostaining and quantitative analysis of rod-bipolar cell axon terminals (*box3*) in control and Ins2^Akita^ mice at different ages. Loss of rod-bipolar cell axon terminals (open arrowheads) in Ins2^Akita^ retinas. (E, J, O) n≥20 retinal images per strain/age, *P<0.05, **P<0.01. 2-way ANOVA. ONL, outer nuclear layer; OPL, outer plexiform layer; INL, inner nuclear layer; IPL, inner plexiform layer; GCL, ganglion cell layer. Scale bar: 10 µm.

The fact that postsynaptic dendrite reduction outweighs presynaptic ribbon loss in the Ins2^Akita^ mouse suggests that the impairment in OPL connectivity may result from the loss of second-order neurons. Thus, we evaluated cell densities of horizontal, cone-bipolar and rod-bipolar cells (Figure 6). Surprisingly, no differences were observed in the density of horizontal, cone-bipolar or rod-bipolar cells between Ins2^Akita^ mice and controls in all age groups (Figure 6E, J). Moreover, quantitative analysis of the total number of cone-bipolar cell axon terminals (Fig. 6K-L) revealed no difference between control and Ins2^Akita^ mice (data not shown). However, a progressive reduction of rod-bipolar cell axonal boutons was observed in the Ins2^Akita^ mouse from 6 months onwards (Figure 6M-O; 6 months, p<0.05; 9 months, p<0.01).

### Changes in amacrine and retinal ganglion cells

**Figure 7:**
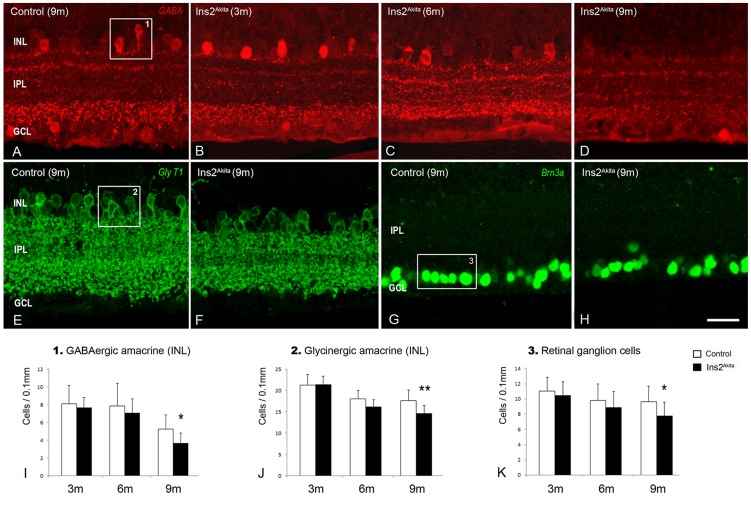
Amacrine and retinal ganglion cell loss in the Ins2^Akita^ mouse. Inner retina photomicrographs of GABA (A-D), GlyT1 (E, F) or Brn3a immnoreactivities (G, H) in 9-month old control and 3-, 6- and 9-month old Ins2^Akita^ mice. Quantitative analysis of INL-GABAergic amacrine cells (*box1*) (I), INL-glycinergic amacrine cells (*box2*) (J) and retinal ganglion cells (*box3*) (K) in control and Ins2^Akita^ mice at different ages. (I-K) n≥20 retinal images per strain/age, *P<0.05, **P<0.01. 2-way ANOVA. INL, inner nuclear layer; IPL, inner plexiform layer; GCL, ganglion cell layer. Scale bar: 20 µm.

To further understand retinal neuropathy in Ins2^Akita^ mice, we examined amacrine cells and RGCs (Fig. 7). Amacrine cells of the INL were assessed by using antibody against GABA or GlyT1 (GABAergic or glycinergic subtypes respectively), since most amacrine cells express one of these neurotransmitters [28], [29]. No differences were observed in either GABAergic or glycinergic amacrine cells between 3-month old controls and Ins2^Akita^ mice (Figure 7I-J). Although the number of GABAergic amacrine cells remained similar to controls by 6 months of age, GABA immunoreactivity was increased at the IPL (Figure 7C). By 9 months, both the density of GABAergic amacrine cells and GABA immunoreactivity were reduced in Ins2^Akita^ mice compared to controls (Figure 7D, 7I). The morphology of glycinergic amacrine cells remained unchanged in control and Ins2^Akita^ mice of all ages (Figure 7E-F). However, there was an age-dependent reduction in the cell density in both strains of mice, although the reduction was greater in the Ins2^Akita^ mouse (Figure 7J). In addition, we observed a 20% reduction in the number of Brn3a^+^ RGCs in 9-month old Ins2^Akita^ mice compared to age-matched controls (p<0.05, Figure 7G-H, K).

## Discussion

This study provides a detailed time-course analysis of the retinal neurodegenerative changes occurring in Ins2^Akita^ mice. Mild scotopic ERG abnormalities and cone photoreceptor degeneration were observed at the early stages of diabetes, followed by significant ERG defects, severe synaptic structure disruption (particularly at the OPL) and loss of amacrine cells and RGCs after long-term diabetes.

Early thinning of the neurosensory retina occurs predominately at the outer retinal layers in Ins2^Akita^ mice, whilst the inner retina progressively degenerates from 3 months onwards. Recent studies reported no changes in retinal thickness in 14–25 weeks old Ins2^Akita^ mice by OCT investigation [30], [31]. What causes the discrepancy is not clear, but it may be related to different approaches in image analysis. The total retinal thickness in those studies was automatically measured between the ILM and Bruch's membrane (BM) using the Heidelberg Eye Explore system. The BM in mouse eyes is not always as clear as other retinal layers. Therefore, we used the OS as a landmark to measure the thickness manually. Our results are supported by previous studies using post-mortem histological investigations [19], [32], which demonstrated significant retinal thinning by 6 months of age. The results are also supported by other data from our study, including neuronal degeneration and abnormal ERG responses.

Diabetes can promote photoreceptor degeneration. Unlike the STZ-induced diabetic rat, in which both cones and rods undergo degeneration [13], [14], the Ins2^Akita^ mouse only develops cone degeneration starting from 3 months and progresses further by 9 months old (which was supported by the abnormal photopic ERG responses observed in this study (Figure S1D) as well as in previous studies [20], [33]). The lack of significant changes in photopic ERG in 3 months old Ins2^Akita^ mice (compared to a significant loss of cone photoreceptors at this stage) may suggest that the traditional white-flash ERG is not sensitive enough to detect this level of neurodegeneration. The absence of significant anomalies in rod photoreceptors together with the large rod/cone ratio (∼30:1) in the mouse retina [34], [35] may explain the relatively minor changes in scotopic a-wave amplitudes occurring in Ins2^Akita^ mice. Cone dysfunctions, including abnormal photopic ERG [36] and impaired color vision sensitivities [12], [37] have been observed in early stages of human diabetes (before the development of clinically detectable DR). In this regard, the Ins2^Akita^ mouse is an excellent model that recapitulates this early neuropathological feature of human DR. Why cone photoreceptors selectively degenerate in the diabetic retina remains elusive. A previous study has shown that cone but not rod metabolism is enhanced with increasing glucose levels in diabetes [38], which may result in excessive oxidative stress to the cells.

Another important observation of this study is the disruption of synaptic structures at the OPL after long-term diabetes, in particular those related to postsynaptic second-order neurons. This is supported by functional ERG changes (significant reduction of the b/a wave ratio) and greater depletion of postsynaptic dendrites compared to presynaptic ribbons. It should be pointed out that the reduced immunoreactivity of calbindin and PKCα at the OPL is likely due to the loss of dendritic and synaptic structures rather than the lack of protein expression, as calbindin and PKCα proteins were detected at high levels in other compartments of horizontal and rod-bipolar cells. Further study using immuno-electron microscopy may help to confirm this. Our results suggest that long-term diabetes can damage synaptic connectivity in the OPL, by preferentially affecting synaptic structures in second-order neurons. Previous studies have observed diverse OPL anomalies in the diabetic eye, although the majority of these are related to photoreceptor presynaptic structures. For example, down-regulation of photoreceptor presynaptic proteins has been observed in rodent models of diabetes [15], [39], whilst mitochondria degeneration was reported in OPL-terminals of photoreceptors and second-order neurons of STZ-diabetic rats [13]. Gao et al. [40] reported a reduction in OPL thickness and OCT-reflectance in patients with mild DR. It has been proposed that the collapse of synaptic structures may be related to the generation of reactive oxygen species [41], [15], a condition that is particularly aggravated in 9-month old Ins2^Akita^ mice [33]. Furthermore, previous studies have shown that hyperglycemic stress can also impair synaptic function in the nervous system [42], [43]. The disruption of retinal synaptic structure in the OPL may be related to the toxic effect of hyperglycemia and oxidative stress to second-order neurons.

DR related retinal neuropathy has previously been linked to the degeneration and dysfunction of inner retinal neurons, including amacrine cells and RGCs [17], [19], [44]. We detected a significant reduction of amacrine cells and RGCs in 9 months old Ins2^Akita^. Previous studies by Gastinger et al. [17], [18] reported a loss of cholinergic and dopaminergic amacrine cells in 6 months old Ins2^Akita^ and a significant degeneration of RGCs in the peripheral retina after 3 months of diabetes. The discrepancy may be related to different immunohistochemical approaches (retinal flatmount versus retinal section) and different cell markers used for morphometry. In the current study, the changes in GABA^+^ amacrine cells in Ins2^Akita^ mice correlate to OPs electrophysiological responses. OPs are known to be a useful tool for evaluating inner retinal activity, particularly amacrine cells [45], [46], and has been used as a reliable parameter to predict the progression of DR [47]. We reported increased OPs responses in Ins2^Akita^ mice by 6 months of age. The enhanced OPs may be related to abnormal expression of GABA in the IPL. A similar change was reported in STZ-diabetic female rats [10]. The turnover of GABA and GABAc receptors is impaired in the diabetic retina [10], [48], and therefore, GABAergic signalling may be compromised. By 9 months of age, amacrine cells were significantly depleted and OPs substantially attenuated.

In summary, we show for the first time that in addition to inner retinal neuronal degeneration, the Ins2^Akita^ mouse presents a variety of retinal neuropathies that are relevant to visual dysfunction in human diabetic patients, including the early reduction of cone photoreceptors, and the disruption of synaptic structures in photoreceptors and second-order neurons. Our findings strongly suggest that the Ins2^Akita^ mouse is a good model for studying diabetic retinal neuropathy.

## Supporting Information

Figure S1 **Scotopic Oscillatory potential (OPs) and phtotopic b-wave responses in the Ins2^Akita^ mouse.** (A, B) Representative OPs responses obtained from 6-month (A) and 9-month old (B), control (black) and Ins2^Akita^ (orange) mice. The 6 individual OPs wavelets are enumerated. (C, D) The amplitude (µV) of the photopic b-wave in control and Ins2^Akita^ mice at 3 (C) and 9 months (D) of age. (C-D) n = 5 mice per strain/age, **P<0.01. 2-way ANOVA.
(TIF)

Figure S2 **Dendritic projections of rod-bipolar and cone-bipolar cells in control and Ins2^Akita^ mice.** Confocal photomicrographs of vertical retinal sections processed for PKCα (A-F) or secretagogin immunoreactivity (G-L) in control and Ins2^Akita^ retinas at 3, 6 and 9 months of age. Control retinas present an age-dependent increment in the length of cone- and rod-bipolar dendritic projections. OPL, outer plexiform layer; INL, inner nuclear layer. Scale bar: 10 µm.
(TIF)

## References

[1] Klein BE (2007) Overview of epidemiologic studies of diabetic retinopathy. Ophthalmic Epidemiol 14: 179–83. 17896294 10.1080/09286580701396720

[2] Lieth E, Gardner TW, Barber AJ, Antonetti DA (2000) Penn State Retina Research Group (2000) Retinal neurodegeneration: early pathology in diabetes. Clin Experiment Ophthalmol 28: 3–8. 11345341 10.1046/j.1442-9071.2000.00222.x

[3] Gardner TW, Antonetti DA, Barber AJ, LaNoue KF, Levison SW (2002) Diabetic retinopathy: more than meets the eye. Surv Ophthalmol 2: S253–62. 10.1016/s0039-6257(02)00387-9 12507627

[4] Antonetti DA, Lieth E, Barber AJ, Gardner TW (1999) Molecular mechanisms of vascular permeability in diabetic retinopathy. Semin Ophthalmol 14: 240–8. 10758225 10.3109/08820539909069543

[5] Leal EC, Manivannan A, Hosoya K, Terasaki T, Cunha-Vaz J, et al (2007) Inducible nitric oxide synthase isoform is a key mediator of leukostasis and blood-retinal barrier breakdown in diabetic retinopathy. Invest Ophthalmol Vis Sci 48: 5257–65. 17962481 10.1167/iovs.07-0112

[6] Wolter JR (1961) Diabetic retinopathy. Am J Ophthalmol 51: 1123–41. 13786453 10.1016/0002-9394(61)91802-5

[7] Bloodworth JM Jr (1962) Diabetic retinopathy. Diabetes 11: 1–22. 13870120

[8] Barber AJ, Gardner TW, Abcouwer SF (2011) The significance of vascular and neural apoptosis to the pathology of diabetic retinopathy. Invest Ophthalmol Vis Sci 52: 1156–63. 21357409 10.1167/iovs.10-6293 PMC3053099

[9] Barber AJ (2003) A new view of diabetic retinopathy: a neurodegenerative disease of the eye. Prog Neuropsychopharmacol Biol Psychiatry 27: 283–90. 12657367 10.1016/S0278-5846(03)00023-X

[10] Ramsey DJ, Ripps H, Qian H (2006) An electrophysiological study of retinal function in the diabetic female rat. Invest Ophthalmol Vis Sci 47: 5116–24. 17065533 10.1167/iovs.06-0364

[11] Roy MS, Gunkel RD, Podgor MJ (1986) Color vision defects in early diabetic retinopathy. Arch Ophthalmol 104: 225–8. 3484948 10.1001/archopht.1986.01050140079024

[12] Trick GL, Burde RM, Gordon MO, Santiago JV, Kilo C (1988) The relationship between hue discrimination and contrast sensitivity deficits in patients with diabetes mellitus. Ophthalmology 95: 693–8. 3262851 10.1016/s0161-6420(88)33125-8

[13] Park SH, Park JW, Park SJ, Kim KY, Chung JW, et al (2003) Apoptotic death of photoreceptors in the streptozotocin-induced diabetic rat retina. Diabetologia 46: 1260–8. 12898017 10.1007/s00125-003-1177-6

[14] Szabadfi K, Atlasz T, Kiss P, Reglodi D, Szabo, et al (2012) Protective effects of the neuropeptide PACAP in diabetic retinopathy. Cell Tissue Res 348: 37–46. 22350850 10.1007/s00441-012-1349-0

[15] Ozawa Y, Kurihara T, Sasaki M (2011) Neural degeneration in the retina of the streptozotocin-induced type 1 diabetes model. Exp Diabetes Res 2011: 108328. 22144984 10.1155/2011/108328 PMC3226536

[16] Preet A, Siddiqui MR, Taha A, Badhai J, Hussain ME, et al (2006) Long-term effect of Trigonella foenum graecum and its combination with sodium orthovanadate in preventing histopathological and biochemical abnormalities in diabetic rat ocular tissues. Mol Cell Biochem 289: 137–47. 16718375 10.1007/s11010-006-9156-0

[17] Gastinger MJ, Kunselman AR, Conboy EE, Bronson SK, Barber AJ (2008) Dendrite remodeling and other abnormalities in the retinal ganglion cells of Ins2 Akita diabetic mice. Invest Ophthalmol Vis Sci 49: 2635–42. 18515593 10.1167/iovs.07-0683

[18] Gastinger MJ, Singh RS, Barber AJ (2006) Loss of cholinergic and dopaminergic amacrine cells in streptozotocin-diabetic rat and Ins2Akita-diabetic mouse retinas. Invest Ophthalmol Vis Sci 47: 3143–50. 16799061 10.1167/iovs.05-1376

[19] Barber AJ, Antonetti DA, Kern TS, Reiter CE, Soans RS, et al (2005) The Ins2Akita mouse as a model of early retinal complications in diabetes. Invest Ophthalmol Vis Sci 46: 2210–8. 15914643 10.1167/iovs.04-1340

[20] Han Z, Guo J, Conley SM, Naash MI (2013) Retinal angiogenesis in the Ins2 (Akita) mouse model of diabetic retinopathy. Invest Ophthalmol Vis Sci 54: 574–84. 23221078 10.1167/iovs.12-10959 PMC3558298

[21] Yoshioka M, Kayo T, Ikeda T, Koizumi A (1997) A novel locus, Mody4, distal to D7Mit189 on chromosome 7 determines early-onset NIDDM in nonobese C57BL/6 (Akita) mutant mice. Diabetes 46: 887–94. 9133560 10.2337/diab.46.5.887

[22] Paques M, Guyomard JL, Simonutti M, Roux MJ, Picaud S, et al (2007) Panretinal, high-resolution color photography of the mouse fundus. Invest Ophthalmol Vis Sci 48: 2769–74. 17525211 10.1167/iovs.06-1099

[23] Xu H, Koch P, Chen M, Lau A, Reid DM, et al (2008) A clinical grading system for retinal inflammation in the chronic model of experimental autoimmune uveoretinitis using digital fundus images. Exp Eye Res 87: 319–26. 18634784 10.1016/j.exer.2008.06.012

[24] Hombrebueno JR, Luo C, Guo L, Chen M, Xu H (2014) Intravitreal Injection of Normal Saline Induces Retinal Degeneration in the C57BL/6J Mouse. Trans Vis Sci Tech 3: 3. 10.1167/tvst.3.2.3 PMC3969215 24757593

[25] Knott EJ, Sheets KG, Zhou Y, Gordon WC, Bazan NG (2011) Spatial correlation of mouse photoreceptor-RPE thickness between SD-OCT and histology. Exp Eye Res 92: 155–60. 21035444 10.1016/j.exer.2010.10.009 PMC3169798

[26] Hombrebueno JR, Tsai MM, Kim HL, De Juan J, Grzywacz NM, et al (2010) Morphological changes of short-wavelength cones in the developing S334ter-3 transgenic rat. Brain Res 1321: 60–6. 20114037 10.1016/j.brainres.2010.01.051 PMC2855539

[27] Chen M, Hombrebueno JR, Luo C, Penalva R, Zhao J, et al (2013) Age- and light-dependent development of localised retinal atrophy in CCL2(−/−)CX3CR1(GFP/GFP) mice. PLoS One 8: e61381. 23637822 10.1371/journal.pone.0061381 PMC3630229

[28] Vaney DI, Young HM (1988) GABA-like immunoreactivity in cholinergic amacrine cells of the rabbit retina. Brain Res 438: 369–73. 3345446 10.1016/0006-8993(88)91366-2

[29] Rice DS, Curran T (2000) Disabled-1 is expressed in type AII amacrine cells in the mouse retina. J Comp Neurol 424: 327–38. 10906706 10.1002/1096-9861(20000821)424:2<327::aid-cne10>3.0.co;2-6

[30] Vagaja NN, Binz N, McLenachan S, Rakoczy EP, McMenamin PG (2013) Influence of endotoxin-mediated retinal inflammation on phenotype of diabetic retinopathy in Ins2 Akita mice. Br J Ophthalmol 97: 1343–50. 23913246 10.1136/bjophthalmol-2013-303201

[31] McLenachan S, Chen X, McMenamin PG, Rakoczy EP (2013) Absence of clinical correlates of diabetic retinopathy in the Ins2Akita retina. Clin Experiment Ophthalmol 41: 582–92. 23433122 10.1111/ceo.12084

[32] Smith SB, Duplantier J, Dun Y, Mysona B, Roon P, et al (2008) In vivo protection against retinal neurodegeneration by sigma receptor 1 ligand (+)-pentazocine. Invest Ophthalmol Vis Sci 49: 4154–61. 18469181 10.1167/iovs.08-1824 PMC2562718

[33] Murray AR, Chen Q, Takahashi Y, Zhou KK, Park K, et al (2013) MicroRNA-200b downregulates oxidation resistance 1 (Oxr1) expression in the retina of type 1 diabetes model. Invest Ophthalmol Vis Sci 54: 1689–97. 23404117 10.1167/iovs.12-10921 PMC3626515

[34] Carter-Dawson LD, LaVail MM (1979) Rods and cones in the mouse retina. I. Structural analysis using light and electron microscopy. J Comp Neurol 188: 245–62. 500858 10.1002/cne.901880204

[35] Carter-Dawson LD, LaVail MM (1979) Rods and cones in the mouse retina. II. Autoradiographic analysis of cell generation using tritiated thymidine. J Comp Neurol 188: 263–72. 500859 10.1002/cne.901880205

[36] Chen H, Zhang M, Huang S, Wu D (2008) The photopic negative response of flash ERG in nonproliferative diabetic retinopathy. Doc Ophthalmol 117: 129–35. 18214565 10.1007/s10633-008-9114-0

[37] Tregear SJ, Knowles PJ, Ripley LG, Casswell AG (1997) Chromatic-contrast threshold impairment in diabetes. Eye (Lond) 11: 537–46. 9425421 10.1038/eye.1997.140

[38] Kurtenbach A, Mayser HM, Jägle H, Fritsche A, Zrenner E (2006) Hyperoxia, hyperglycemia, and photoreceptor sensitivity in normal and diabetic subjects. Vis Neurosci 23651–61 10.1017/S095252380623339X 16962009

[39] VanGuilder HD, Brucklacher RM, Patel K, Ellis RW, Freeman WM, et al (2008) Diabetes downregulates presynaptic proteins and reduces basal synapsin I phosphorylation in rat retina. Eur J Neurosci 28: 1–11. 18662330 10.1111/j.1460-9568.2008.06322.x

[40] Gao W, Tátrai E, Ölvedy V, Varga B, Laurik L, et al (2011) Investigation of changes in thickness and reflectivity from layered retinal structures of healthy and diabetic eyes with optical coherence tomography. J Biomed Sci Eng 4: 657–665.

[41] Kowluru RA, Chan PS (2007) Oxidative stress and diabetic retinopathy. Exp Diabetes Res 2007: 43603. 17641741 10.1155/2007/43603 PMC1880867

[42] Kamal A, Biessels GJ, Ramakers GM, Hendrik Gispen W (2005) The effect of short duration streptozotocin-induced diabetes mellitus on the late phase and threshold of long-term potentiation induction in the rat. Brain Res 1053: 126–30. 16038887 10.1016/j.brainres.2005.06.036

[43] Yamato T, Misumi Y, Yamasaki S, Kino M, Aomine M (2004) Diabetes mellitus decreases hippocampal release of neurotransmitters: an in vivo microdialysis study of awake, freely moving rats. Diabetes Nutr Metab 17: 128–36. 15334789

[44] Seki M, Tanaka T, Nawa H, Usui T, Fukuchi T, et al (2004) Involvement of brain-derived neurotrophic factor in early retinal neuropathy of streptozotocin-induced diabetes in rats: therapeutic potential of brain-derived neurotrophic factor for dopaminergic amacrine cells. Diabetes 53: 2412–9. 15331553 10.2337/diabetes.53.9.2412

[45] Wachtmeister L, Dowling JE (1978) The oscillatory potentials of the mudpuppy retina. Invest Ophthalmol Vis Sci 17: 1176–88. 721390

[46] Wachtmeister L (1998) Oscillatory potentials in the retina: what do they reveal. Prog Retin Eye Res 17: 485–521. 9777648 10.1016/s1350-9462(98)00006-8

[47] Tzekov R, Arden GB (1999) The electroretinogram in diabetic retinopathy. Surv Ophthalmol 44: 53–60. 10466588 10.1016/s0039-6257(99)00063-6

[48] Ishikawa A, Ishiguro S, Tamai M (1996) Changes in GABA metabolism in streptozotocin-induced diabetic rat retinas. Curr Eye Res 15: 63–71. 8631205 10.3109/02713689609017612

