# Supplementary figures and images for: Loss of Synaptic Connectivity, Particularly in Second Order Neurons Is a Key Feature of Diabetic Retinal Neuropathy in the Ins2Akita Mouse
Source: PLoS One. 2014 May 21;9(5):e97970. doi: 10.1371/journal.pone.0097970 (PMC4029784; doi:10.1371/journal.pone.0097970)

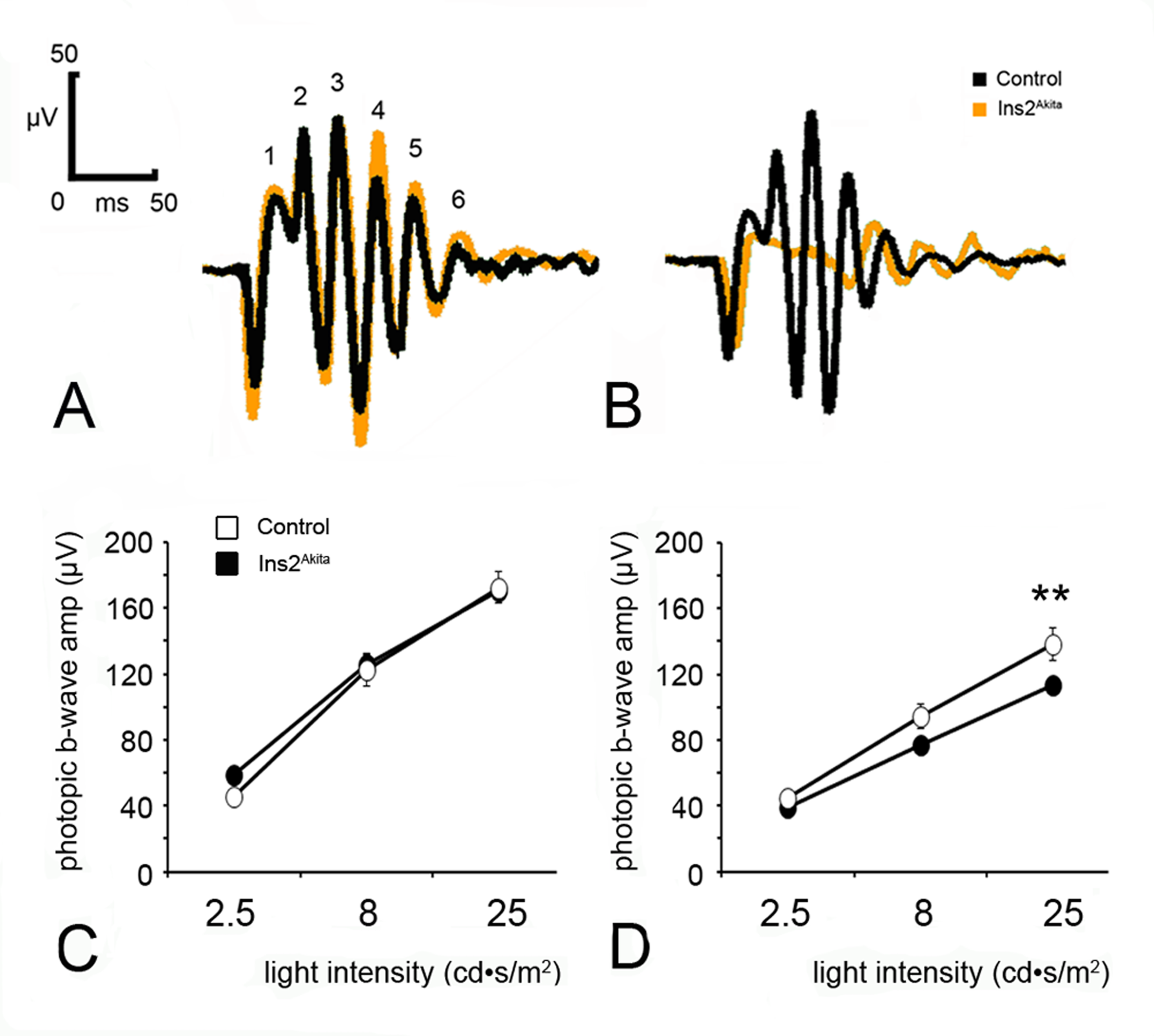

Supplement: Figure S1 — Scotopic Oscillatory potential (OPs) and phtotopic b-wave responses in the Ins2Akita mouse. (A, B) Representative OPs responses obtained from 6-month (A) and 9-month old (B), control (black) and Ins2Akita (orange) mice. The 6 individual OPs wavelets are enumerated. (C, D) The amplitude (µV) of the photopic b-wave in control and Ins2Akita mice at 3 (C) and 9 months (D) of age. (C-D) n = 5 mice per strain/age, **P<0.01. 2-way ANOVA. (TIF) [file pone.0097970.s001.tif]

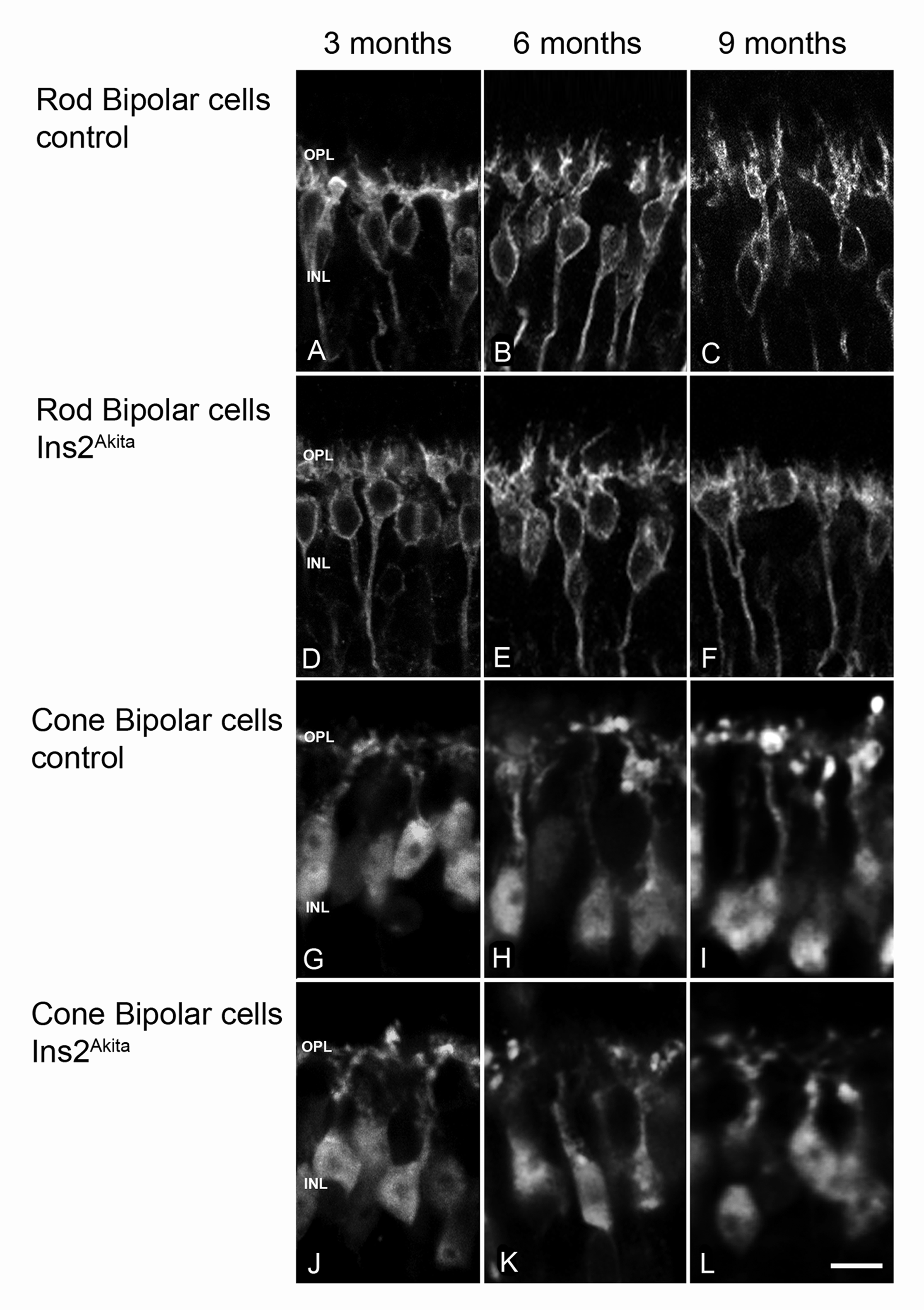

Supplement: Figure S2 — Dendritic projections of rod-bipolar and cone-bipolar cells in control and Ins2Akita mice. Confocal photomicrographs of vertical retinal sections processed for PKCα (A-F) or secretagogin immunoreactivity (G-L) in control and Ins2Akita retinas at 3, 6 and 9 months of age. Control retinas present an age-dependent increment in the length of cone- and rod-bipolar dendritic projections. OPL, outer plexiform layer; INL, inner nuclear layer. Scale bar: 10 µm. (TIF) [file pone.0097970.s002.tif]
